# Calcitonin levels in washout samples vs. cytology in the detection of malignant lymph node metastasis in recurrent medullary thyroid cancer

**DOI:** 10.3906/sag-2105-280

**Published:** 2021-09-12

**Authors:** Asena GÖKÇAY CANPOLAT, Mustafa ŞAHİN, Koray CEYHAN, Serpil SAK, Özgür DEMİR, Rıfat EMRAL, Murat Faik ERDOĞAN, Sevim GÜLLÜ, Demet ÇORAPÇIOĞLU

**Affiliations:** 1 Division of Endocrinology and Metabolism, Department of Internal Medicine, Faculty of Medicine, Ankara University, Ankara, Turkey; 2 Department of Pathology, Faculty of Medicine, Ankara University, Ankara, Turkey

**Keywords:** Recurrent medullary thyroid cancer, fine needle aspiration cytology, calcitonin, washout sample

## Abstract

**Background/aim:**

Calcitonin level in fine-needle aspirate washout fluid (Ct-FNA) was extensively studied for thyroid nodules and lymph nodes (LN). However, the data was scarce for neck recurrences/metastases of medullary thyroid cancer (MTC). Thus, the diagnostic accuracy of Ct-FNA and cytology in the detection of neck LN metastases of recurrent MTC cases were assessed.

**Materials and methods:**

The database of MTC patients between 2010 and 2021 was retrospectively reviewed. A total of 32 patients with recurrent MTC and suspicious LN who underwent FNA and Ct measurement from washout samples were included in this study. Preoperative serum Ct (sCt), Ct-FNA, Ct-FNA/sCt ratio, cytology data were recorded for all participants.

**Results:**

Median sCt of 32 patients and Ct-FNA washout fluid levels of operated suspicious 44 LNs were 723 (54–9000) pg/mL and 1800 (151–9500) pg/mL, respectively. The diagnostic accuracy of Ct-FNA washout fluid was greater than cytology (95.4% vs. 86%, respectively). Using a cut-off level of >638.5 pg/mL, the Ct-FNA predicted the diagnosis of LN metastasis of recurrent MTC with a sensitivity of 80% and specificity of 94.9%. Furthermore, using a cut-off level of >1.16, the Ct-FNA/sCt ratio well predicted the diagnosis of LN metastasis of recurrent MTC with a sensitivity of 92.3% and specificity of 100%.

**Conclusion:**

As Ct-FNA has greater diagnostic accuracy in our study, it would be complementary to cytology results to localize metastatic LNs in recurrent MTC. Furthermore, for the first time, we demonstrated that the Ct-FNA/sCt ratio was a better predictor of metastatic LNs in recurrent MTC than a particular cut-off for Ct-FNA alone.

## 1. Introduction

Medullary thyroid carcinoma (MTC) is a neuroendocrine malignancy, that originates from calcitonin (Ct) producing parafollicular C-cells of the thyroid and accounts for 3%–5% of all thyroid malignancies [1]. Metastasis to regional lymph nodes (LN) may occur more often and earlier than in differentiated thyroid cancers (DTC) [2]. Even if the initial surgery is supposed to be adequate, the recurrence rate remains up to 40%–66% [3]. MTC can metastasize to the cervical LNs in 68%–80% of patients [4]. The biochemical cure is achievable in 18.6% of reoperative patients through the use of compartment-oriented LN microdissection [3]. The optimal surgical management for the persistent or recurrent disease remains controversial, because of the long-term outcomes for these procedures. The 5- and 10-year survival for medullary carcinomas are 65%–89% and 71%–87%, respectively [5] and it handles up to 14% of thyroid-related deaths [6].

Serum Ct is a useful marker in diagnosis, differential diagnosis, assessment of treatment, and follow-up of the MTC [7,8]. But, the location of MTC in the thyroid gland or cervical metastases cannot be achieved by serum Ct measurements. Fine-needle aspiration cytology (FNAC) represents the current diagnostic technique for the identification of MTC neck LN metastases both before and after thyroidectomy. However, the diagnostic accuracy of FNAC (<50%) in diagnosing MTC is not as high as it is for DTCs [9]. Ct measurement in the wash-out fluid of FNA (Ct-FNA) to diagnose MTC, represents an additional highly reliable marker, and it improves the sensitivity of the diagnosis [10]. While there was no such a recommendation before, the recent American Thyroid Association (ATA) MTC guideline declared the usefulness of Ct-FNA when FNA findings are inconclusive or suggestive of MTC [11]. Until now, many studies and metaanalyses have discussed the utility of this method for thyroid nodules and LNs, but due to the rarity of the disease, a few studies have been conducted for neck recurrences/metastases of MTC [1,12].

The purpose of this present study was to compare the clinical usefulness of Ct-FNA versus cytology in the identification of neck LN metastases in our recurrent cases and select a ratio for Ct-FNA/sCt rather than Ct-FNA cut-off values for LN metastases for the first time.

## 2. Materials and methods

MTC patients (between January 2010 and January 2021) were retrospectively analyzed. Clinical data were obtained from medical records of Ankara University, Faculty of Medicine, İbni Sina Hospital. Physical examination, reports of neck ultrasonography (US), cytology results of US-guided FNAC of suspicious LNs, and Ct-FNA results were recorded. The participants signed the written informed consent before the FNAC procedure. The study protocol was in compliance with the principles outlined in the Declaration of Helsinki and approved by the Institutional Ethics Committee (İ11-712-20). Recurrent MTC was defined as evidence of loco regional (either single/or a group of LNs) or distant MTC in which the postoperative sCt level was normal after initial surgery at least 6 months [13]. Reoperations were done according to curative intent or palliative intent.

### 2.1. Assessment of serum calcitonin

Blood samples were obtained from participants in the morning, after 12 h of fasting, to measure serum Ct and carcinoembryonic antigen (CEA) at preoperative and postoperative period. None of the patients had hyperparathyroidism, hepatic or renal insufficiency. None of them were smokers or were using drugs that stimulate Ct secretion.

Serum TSH, fT3, fT4 levels were assessed using a direct chemiluminescence immunoassay (Siemens, ADVIA Centaur XP ImmunoassaySystem, Tarrytown, NY, USA). The Ct levels were measured with immunoradiometric assay (IRMA) (Diasource Immunoassays S.A, catalog number KIP0429, Belgium). Human CEA ELISA Kit was used for the colorimetric detection method (Abcam, catalog number ab99992, United Kingdom). Normal range values for serum Ct are less than <10 pg/mL for both men and women and CEA less than 3 ng/mL for nonsmokers and less than 7 ng/mL for smokers, respectively.

### 2.2. Assessment of the neck and pathological lymph nodes

A group of experienced endocrinologists (M.F.E., S.G., R.E., M.Ş., and A.G.C.) performed the thyroid ultrasonography by using a high-resolution Hitachi EUB 7000 HV machine with a 6–13 MHz linear transducer, Tokyo, Japan. All cervical LNs were identified and localized, and their diameters were measured. Suspicious LNs were identified according to ATA guidelines [14, 15] and submitted to FNAC.

### 2.3. Evaluation of FNAC and Ct-FNAC

Ultrasound-guided FNAC was performed with a 23 or 25-gauge needle and the aspirated material was smeared directly on glass slides and air-dried smears were stained with May-Grunwald-Giemsa (MGG) stain. An experienced thyroid cytopathologist (K.C.), who was unaware of Ct-FNAC results, made the cytological examination. Cytology was reported as positive for malignancy/consistent with MTC metastasis, negative for malignancy/benign cytology and nondiagnostic cytology/unsatisfactory for cytological evaluation.

A total of 44 suspicious LNs were aspirated for Ct-FNAC and FNAC. But because of the paucity of one of the aspirated materials to be smeared to the slides, it had not been sent to the cytology department, and it had been rather used for wash-out sample.

After smearing, the remaining aspirate in the syringe and needle were rinsed with 0.5 mL of normal saline in micro-centrifuge tubes. This washout sample was processed for Ct-FNA measurement. The Ct-FNA levels were measured with immunoradiometric assay (IRMA) (Diasource Immunoassays S.A, Belgium). A Ct-FNA level higher than serum Ct level was considered positive rather than an arbitrary cut-off level likewise earlier reports to eliminate peripheral blood contamination.

### 2.4. Histopathological evaluation

After LN dissection, an expert thyroid-specific pathologist (S.D.S) who is unaware of cytological and Ct-FNA results made histological diagnoses. The histopathological diagnosis of MTC was done with staining with chromogranin, Ct, and/or CEA; and negatively stained with thyroglobulin (Tg) and thyroid transcription factor 1 **(**TTF-1).

## 3. Statistical analysis

The Kolmogorov–Smirnov criterion was used for the assessment of normality. Continuous variables were expressed as a mean+standard deviation or median (minimum and maximum). Discrete variables were expressed as median with minimum and maximum values instead of interquartile range to demonstrate the heterogeneity of data. Categorical variables were summarized as frequencies and percentages. The sensitivity, specificity, diagnostic accuracy of FNAC and Ct-FNA were calculated. Differences in sensitivity, specificity, and diagnostic accuracy between Ct-FNA and FNAC were evaluated by the Fisher’s exact test. The capacity of Ct-FNA and Ct-FNA/sCt values in predicting metastasis in LNs were analyzed using receiver operating characteristic (ROC) curve analysis. When a significant cut-off value was observed, the sensitivity, specificity values were presented. While investigating the associations between Ct-FNA and sCt levels, the correlation coefficients and their significance were calculated using the Spearman test.

Statistical analyses were performed using SPSS statistical software (IBM Corp. Released in 2015. IBM SPSS Statistics for Windows, Version 23.0. Armonk, NY: IBM Corp.). A two-tailed p-value <0.05 was determined as statistically significant.

## 4. Results

A total of 94 patients with the diagnosis of MTC were included. There were 54 women and 40 men with a mean age of 40.7 ± 17 years. A total of 61 cases (65%) were sporadic while, 30 cases (32%) were multiple endocrine neoplasia (MEN) type 2A and 3 (3%) cases were MEN 2B.

The median sCt level was 620 (54–9000) pg/mL, the median CEA level was 9.4 (0.5–1958) ng/mL, and the mean MTC lesion size in the thyroid was 26 ± 12.9 mm. Most of the MTC histopathology was reported as multiple foci at 27 patients (29%). Then, the MTC lesions were almost evenly disturbed in the right (n = 18, 19%) and the left (n = 14, 15%) lobes, and less frequently as C-cell hyperplasia (n = 4, 4%). The first operation and histopathological evaluation of the 31 cases (n = 33%) were done in another center.

Overall, 32 patients (F/M: 15/17) were recurrent cases with cervical LN/distant metastasis for MTC. The preoperative median sCt level in recurrent cases was 723 (54–9000) pg/mL and the preoperative CEA level was 9 (0.5–1958) ng/mL. FNAC was performed on 43, and Ct-FNAC was performed on 44 suspicious LNs. The median largest diameter of the LNs was 9 (4–16) mm. The most frequent localization of the regional recurrences of the neck were found as left thyroid bed, right level III, and IV of the lateral neck, respectively.

All 32 patients underwent surgery. Some patients (n: 5) had predominant distant metastasis such as liver (n: 2), lung (n: 2), or bone (n: 1) at the time of cervical reoperation which were detected with neck/thorax/abdomen computerized tomography (CT) and bone scintigraphy. Tumor debulking was done for the neck due to airway/esophageal obstruction (with multiple neck metastasis of LNs) or alleviating the diarrheal syndrome. The remaining patient who had highly elevated basal sCt levels (n: 10), had undergone the same imaging procedure but no involvement other than neck had been detected. The distribution of the surgeries was as follows, tumor debulking and LN dissection for 2 patients with a total of 5 LNs, only single neck LN metastasis for 18 patients for 18 LNs, and lymph node dissection for 12 patients with a total of 21 LNs.

The postoperative pathological examination revealed 42 LN compatible with MTC and 2 benign (reactive) LNs. FNAC findings for these LNs (Table 1) and the histopathological distribution according to the FNAC findings were described in Table 2. FNAC had a specificity of 100% and a sensitivity of 85.3%.

The median Ct-FNA level of operated suspicious 44 LN was 1800 (151–9500) pg/mL. 42 LNs, all with positive Ct-FNA, were confirmed as MTC histopathologically. 2 of them with Ct-FNA results of 800 and 221 pg/mL were diagnosed as benign (reactive) LNs.

Ct-FNA levels were same between sporadic 1507 (151–9500) pg/mL and MEN 2A/2B cases 1800 (182–7154) pg/mL. There was not a correlation between Ct-FNA and sCt levels (p: 0.07, r: 0.34).

The diagnostic accuracies of FNAC versus Ct-FNA were found 86% and 95.4%, respectively.

The median ratio of Ct-FNA/sCt levels for the entire group, sporadic cases, MEN2A and MEN2B (n: 1) were 2.5 (1.06–28.1); 2.3 (1.06–23.6); 2.5 (1.3–28) and 6, respectively. The tumor localizations of hereditary cases were 11% left lobe and 89% multiple foci for MEN2A, and 100% multiple foci for MEN2B. These multiple foci were predominantly made up of areas of C-cell hyperplasia.

ROC curve analysis showed an area under the curve (AUC): 97%, (p: 0.001) for Ct-FNA, and the cut-off >638.5 pg/mL was determined according to the analysis with a sensitivity of 80% and specificity of 94.9%. Based on the curve, the AUC was 98% (p: 0.001) and the cut-off value of Ct-FNA/sCt ratio was > 1.16 leading to 92.3% sensitivity and 100% specificity.

## 5. Discussion

The primary aim of this study was to compare FNAC and Ct-FNA results. For this purpose, there were studies involving combined analysis of nodules and LNs together previously. There was limited knowledge about the comparison of FNAC and Ct-FNA in recurrent metastatic LNs of MTC. In this retrospective study, we evaluated 43 FNAC and 44 Ct-FNA results only in recurrent suspicious MTC LNs in a series of 32 patients for the first time. Our major findings for metastatic LNs of MTC were cut-off level for Ct-FNA was >638.5 pg/mL, Ct-FNA/sCt ratio was >1.16, and diagnostic accuracy of Ct-FNA was higher than FNAC (95.4% versus 86%).

As the survival varies by the extent of local disease, the determination of the extent of the disease and the diagnosis of the metastatic LNs with preoperative FNAC, Ct-FNA procedures promise to achieve successful surgical outcomes in recurrent cases of MTC.

Even though serum Ct is the most sensitive diagnostic tool for MTC and has higher sensitivity compared to cytology [16]; it does not point out the localization of the primary tumor/metastases or recurrences in the neck after initial surgery [1,17]. Also, it may be increased owing to conditions such as renal failure, hypercalcemia, cigarette smoking, neuroendocrine tumors, hypergastrinemia, increased age, and interference with heterophilic antibodies [18–20].

FNAC is the safe, accurate, and cost-effective approach for the detection of metastatic LNs [21]. But the diagnosis of metastatic LNs of MTC depends on the knowledge and experience of the cytopathologist because its diagnostic accuracy is not as high as it is for PTC [8]. According to a metaanalysis, the detection rate of FNAC in patients with MTC was between 12.5% and 88.2%, with a pooled estimate of 56.4% [20]. The major reasons for the low detection rate of MTC with FNAC were inadequate sampling, poor cellularity, nontypical cell shapes, and unusual cytomorphologic presentations [22]. MTC was also called a great mimicker owing to its many possible microscopic patterns [19,23].

So, Ct-FNA is an ancillary diagnostic tool that can provide useful information both for diagnosis and localization of the tumor. After the first reports from Boi et al. and Kudo et al., several reports showed that Ct-FNA supported and increased the accuracy of cytopathologic diagnosis[1, 7, 8, 10, 18, 24]. Finally, the ATA MTC guideline revised the suggestion as “Ct-FNAC should accurately diagnose MTC and avoid false-negative or inconclusive/nondiagnostic results from cytology [11].”

Likewise, in previous findings, we found higher diagnostic accuracy (95.4% versus 86%) but lower specificity (80% versus 100%) belonging to Ct-FNA for a cut-off >638.5 than FNAC results. The diagnosis of MTC can not be solely made with Ct-FNA, but be integrated with FNAC. Two of the LNs were false positive with Ct-FNA and two of the LNs were false negative with FNAC, so Ct-FNA is an additional procedure and can not substitute FNAC. The combined usage of the two methods provides better accuracy than any of them alone.

There was no correlation between Ct-FNA and sCt levels. It was expected as a result of blood contamination, but if there is no/little blood contamination or punction (FNAC) is successful, the correlation between them could not be obtained. Also, there is not any knowledge in the literature showing a correlation between the size of the lymph node and Ct-FNA. Moreover, there are differences in the respective amounts of Tg and Ct within the metastatic cells or released into the interstitial fluid during needle aspiration.

The main limitation of the previous studies was how to interpret the results of Ct-FNA correctly, as it is an obligation to establish a cut-off value for Ct-FNA to exclude the effect of peripheral blood contamination, especially in patients with extremely high sCt. Giovanella et al. and Trimboli et al. also stated the importance of pre-analytic issues such as different Ct assays, the inter-method variability, and sample preparation for Ct-FNA [25, 26]. We selected the ratio rather than an arbitrary cut-off to establish a better diagnostic accuracy as it had been suggested for MTC nodules and parathyroid adenomas [7, 27]. We found a Ct-FNA/sCt ratio greater than 1.16 with a sensitivity of 92.3 and specificity of 100%, which promises a more accurate diagnosis compared to fixed cut-off ratios for metastatic LNs. The median ratio was 2.5 (1.06–28.1) for the entire group. The cut-off was much lower than reported for differentiated thyroid cancers and wash-out levels for thyroglobulin, but this was explained due to differences in the respective amounts of Tg and Ct within the metastatic cells or released into the interstitial fluid during needle aspiration[1].

The limitations of our study were a small sample size for recurrent LN metastasis because of the rarity of the situation. Patients with high sCt levels and peripheral contamination risk were other limitations. This study had some strengths, including being the only study with the highest number of lymph nodes with recurrent LNs, and comparisons of FNAC, sCt, and Ct-FNA/sCt for LNs especially in cases of markedly elevated sCt levels for the first time.

In conclusion, Ct-FNAC, as an adjunct to FNAC, should be performed as a preoperative surgical localization technique to avoid false-negative MTC in all patients with suspicious MTC. We suggest that the usage of a ratio rather than a particular cut-off level will give more accurate results, especially when markedly elevated sCt values are measured.

## Figures and Tables

**Table 1:** Fine needle aspiration cytology findings of the suspicious lymph nodes.

	Total	Hereditary	Sporadic
Nondiagnostic	5 (11.6%)	3 (7%)	2 (4.6%)
Benign	2 (4.6%)	1 (2.3%)	1 (2.3%)
Suspicious for malignancy	1 (2.2%)	1 (2.3%)	-
MTC	35 (81.3%)	13 (30%)	22 (51%)

MTC: Medullary Thyroid Cancer.

**Table 2:** Histopathological distribution of fine needle aspiration cytology (FNAC) and calcitonin level in fine-needle aspirate washout fluid (Ct-FNA).

Histopathological results	FNAC results	Ct-FNA (pg/mL)
Benign LN	2 nondiagnostic	800 and 221
MTC LN	35 MTC	1800 (151–9500)
	3 nondiagnostic	1800, 1800, 800
	2 bening LN	2492 and 800
	1 suspicious for malignancy	1700

LN: Lymph Node, MTC: Medullary Thyroid Cancer.
